# Perceptions and experiences of the mistreatment of women during childbirth in health facilities in Guinea: a qualitative study with women and service providers

**DOI:** 10.1186/s12978-016-0266-1

**Published:** 2017-01-11

**Authors:** Mamadou Diouldé Balde, Boubacar Alpha Diallo, Abou Bangoura, Oumar Sall, Anne Marie Soumah, Joshua P. Vogel, Meghan A. Bohren

**Affiliations:** 1 Cellule de recherche en santé de la reproduction en Guinée (CERREGUI), University National Hospital-Donka, Conakry, Guinea; 2 Faculté de Médecine, Pharmacie et Odontostomatologie, Université G.A.Nasser de Conakry, Conakry, Guinea; 3 Département de sociologie, Université Sonfonia, Conakry, Guinea; 4 UNDP/UNFPA/UNICEF/WHO/World Bank Special Programme of Research, Development and Research Training in Human Reproduction (HRP), Department of Reproductive Health and Research, World Health Organization, Avenue Appia 20, 1211 Geneva, Switzerland

**Keywords:** Mistreatment, Quality of care, Disrespect and abuse, Guinea, Maternal health, Childbirth

## Abstract

**Background:**

Every woman is entitled to respectful care during childbirth; so it is concerning to hear of informal reports of mistreatment during childbirth in Guinea. This study sought to explore the perceptions and experiences of mistreatment during childbirth, from the perspectives of women and service providers, and the analysis presents findings according to a typology of mistreatment during childbirth.

**Methods:**

This study used qualitative methods (in-depth interviews (IDIs) and focus group discussions (FGDs)) and was conducted with four groups of participants: women of reproductive age, midwives, doctors, and administrators. The study took place in two sites in Guinea, an urban area (Mamou) and peri-urban (Pita). Data collection was conducted in two health facilities for providers and administrators, and in the health facility catchment area for women. Data were collected in local languages (Pular and Malinké), then transcribed and analyzed in French. We used a thematic analysis approach and coded transcripts manually.

**Results:**

A total of 64 IDIs and eight FGDs were conducted and are included in this analysis, including 40 IDIs and eight FGDs with women of reproductive age, 5 IDIs with doctors, 13 IDIs with midwives, and 6 IDIs with administrators. Participants described their own personal experiences, experiences of women in their communities and perceptions regarding mistreatment during childbirth. Results were organized according to a typology of mistreatment during childbirth, and included instances of physical abuse, verbal abuse, abandonment and neglect. Women described being slapped by providers, yelled at for noncompliance with provider requests, giving birth on the floor and without skilled attendance in the health facility. Poor physical conditions of health facilities and health workforce constraints contributed to experiences of mistreatment.

**Conclusions:**

These results are important because they demonstrate that the mistreatment of women during childbirth exists in Guinea and occurs in multiple forms. These data should be used by the Ministry of Health and other stakeholders to develop strategies to reduce and prevent the mistreatment of women during childbirth.

## Plain English summary

Over 300,000 maternal deaths occurred globally in 2015, with approximately two-thirds occurring in sub-Saharan Africa. Most maternal deaths can be avoided by increasing the number of births occurring with skilled healthcare providers and number of births in well-equipped hospitals. Improving the quality of care provided to women and their babies during the childbirth period is therefore an important part of improving women’s health. However, several challenges remain in Guinea, a country in West Africa, where limited health resources exist, drugs and medical supplies are unreliable, and the number of doctors, midwives and nurses is insufficient to cover the needs of the people. Anecdotal reports from women and healthcare providers in Guinea suggest that women may be mistreated during childbirth, such as being pinched, slapped, and verbally abused. This study was conducted in two areas of Guinea, and used qualitative methods (in-depth interviews and focus group discussions) to explore how women and healthcare providers in Guinea experience and perceive mistreatment during childbirth. Qualitative methods allowed us to hear from the voices of women and healthcare providers themselves, as they shared their own personal experiences, experiences of women in their communities and perceptions regarding mistreatment during childbirth. Women described being slapped by healthcare providers, yelled at for not complying with healthcare provider requests, giving birth on the floor and without skilled attendance in the health facility. These results will be used by the Ministry of Health and other stakeholders to develop strategies to reduce and prevent the mistreatment of women during childbirth.

## Background

An estimated 303,000 maternal deaths occurred globally in 2015, with approximately two-thirds occurring in sub-Saharan Africa [1]. In 2000, the global community started an ambitious program to improve health and development through the Millennium Development Goals (MDGs). Target 5 aimed to improve maternal health, by reducing the maternal mortality ratio (MMR, maternal deaths per 100,000 live births) by 75 percent by 2015 and achieving universal access to reproductive health services [1]. Key aspects of reducing maternal mortality include increasing skilled birth attendance and childbirth in health facilities that are adequately equipped with trained personnel, physical resources, medical supplies, and can provide emergency obstetric and newborn care [2, 3]. Improving quality of care during the childbirth period is therefore an important step in improving maternal health. However, key challenges remain in Guinea, where limited physical resources exist in the health facilities, medical and drug supply chains are unreliable, health worker shortages exist, and the cost of care can be prohibitively expensive [4, 5].

Given the global efforts to improve maternal health and quality of care, the issue of mistreatment of women during childbirth has received increasing attention in the past few years. Similarly, improving women’s *experiences of care* has been identified by the World Health Organization as an critical component of strategies to improve quality of care [6]. This includes respectful care for the woman, effective communication between the provider and the woman, and emotional support for the woman during labor and childbirth [6]. A recent systematic review synthesized global evidence on mistreatment during childbirth, and presented a new typology to describe the phenomenon [7]. This review built on other work in the field, including a landscape analysis [8] and primary studies in Kenya, Tanzania and Nigeria [9–11]. In Guinea, anecdotal evidence suggests that women across the country experience mistreatment during childbirth. However, to date there has been no research or programs to address this issue. Research on mistreatment is clearly a necessary step to explore and understand what is happening to women, work with providers to change their behaviors, and to prevent mistreatment from occurring.

This study is part of a multi-country study on mistreatment of women during childbirth in four countries: Guinea, Ghana, Nigeria and Myanmar [12]. The first phase of the study is qualitative, aiming to better understand factors contributing to mistreatment during childbirth and identify areas where interventions could be developed. The second phase is a measurement phase, and the measurement tools will be informed from the findings of the qualitative phase. In Guinea, this study was carried out in Mamou and Pita. Mamou is in central Guinea, approximately 300 km from Conakry, and has 30,982 inhabitants. There is one regional hospital and five health facilities. Pita is in the Mamou Region and has 18,676 inhabitants.

This paper presents the qualitative findings on perceptions and experiences of mistreatment of women during childbirth in health facilities in Guinea. The topic was chosen to present the existence of mistreatment during childbirth in Guinea, and the impact that mistreatment can have on the woman’s future healthcare decisions. The findings are presented according to the typology of mistreatment during childbirth developed by Bohren and colleagues [7].

### Overview of maternal health in Guinea

According to 2015 estimates in Guinea, 55.5% of women give birth without a skilled attendant, and a woman’s lifetime risk of maternal death is 1 in 25 [1, 2]. Approximately 40 percent of women in Guinea give birth in a health facility, of which 36 percent give birth in public sector health facility and 5 percent in a private sector health facility [13, 14]. In Mamou, the MMR in 2014 was 1203 per 100,000 live births, compared to Conakry in 2010, 819 per 100,000 live births [4, 5]. Guinea has been impacted by the Ebola virus since 2014; however the Mamou region was minimally impacted. Two of the major barriers to improving quality of care during childbirth in the Mamou prefecture is lack of equipment in health facilities and health worker shortages [15]. For example, in Guinea there are approximately 108 obstetrician-gynecologists, 409 midwives and 1189 nurses to serve a population of almost twelve million [15].

## Methods

### Study sites

The two sites selected for this study (Mamou and Pita) are in the same administrative region. Mamou is an urban location with a regional hospital, and Pita is a peri-urban location with a prefectural (district-level) hospital. This study took place in these health facilities, as well as the communities that are within the facility catchment areas. In this region, the skilled birth attendance rate is 20.7 percent [13]. The mean age of first marriage for women is seventeen years, compared to men at 26 years, and the total fertility rate is 5.4 (number of children born per woman), compared to the national fertility rate of 3.8 [13].

### Study participants, recruitment and sampling

Three groups of participants were identified for this study. First, in-depth interviews (IDIs) and focus group discussions (FGDs) were conducted with women of reproductive age (18–49). Inclusion criteria for women of reproductive age is: women with previous experience (previous 1 year for IDIs and previous 5 years for FGDs) of childbirth in a health facility and currently living in the facility catchment area. Community health workers identified women who met the inclusion criteria and helped connect the research assistants in person. Second, IDIs were conducted with midwives, nurses and doctors working on the maternity ward of the study facilities. Third, IDIs were conducted with facility administrators, such as the medical director or matron-in-charge. Both IDIs and FGDs were conducted with women in order to gain a detailed understanding of experiences of mistreatment during childbirth (IDIs) and to better understand social norms related to mistreatment (FGDs). Only IDIs were conducted with providers and administrators, due to concerns that FGDs may breach the confidentiality of study participants through the disclosure of poor practices or “naming and blaming”. All potential participants were invited to participate and provide consent. Participants were recruited until the desired sample size was reached and no new themes were emerging from the data (data saturation).

### Data collection and management

This study used a qualitative approach to data collection, with semi-structured IDI and FGD guides. The discussion guides were similar between IDIs and FGDs and covered these topics: (1) story of childbirth; (2) perceptions and experiences of childbirth occurring in health facilities; (3) elements and experiences of mistreatment during childbirth; (4) perceived factors influencing how women are treated during childbirth; (5) acceptability of scenarios of mistreatment during childbirth. The research team for this study is a group of medical doctors and sociologists affiliated with Cellule du recherche en la sante de la reproduction (CERREGUI). There were 10 data collectors in total, eight women and two men. Before starting data collection, there was a training workshop in Conakry for the research team. During the workshop, the study protocol and discussion guides were discussed in detail. All IDI and FGD discussion guides were pre-tested in order to evaluate, improve and adapt the discussion guides for the Guinea context (from the multi-country study protocol [12]). During data collection, IDIs and FGDs with women were conducted in private, quiet areas in the community, and data collectors were women. IDIs with providers and administrators were conducted in a private room in the health facility. All participants were contacted once. IDIs and FGDs lasted approximately 60 to 90 minutes, and participants received a snack and drink to show appreciation for their time. All IDIs and FGDs were audio recorded, and transcribed verbatim from local language (Pular and Malinke), then translated to French by the research team. Data collection and transcription was four months in duration (June to September 2015).

### Data analysis

We used a thematic analysis approach, as described by Braun and Clark [16]. The analysis process started at an analysis workshop for the study teams from Guinea, Ghana and Nigeria. We used the typology of mistreatment during childbirth proposed by Bohren and colleagues [7] to start building the codebook. The codebook was supplemented with codes emerging from the data and from the discussion guides. Coding was conducted manually using Microsoft Word by two researchers from CERREGUI with medical and sociology training, with the support of the research team. Data collection was conducted primarily in French, and supplemented with Pular and Malinke where appropriate. Key findings were translated into English at the time of manuscript writing. Throughout analysis process, the researchers considered how their worldview and training may influence their interpretation of the results (reflexivity).

## Results

### Overview

A total of 64 IDIs and 8 FGDs were conducted and are included in this analysis, including 40 IDIs and 8 FGDs with women of reproductive age, 5 IDIs with doctors, 13 IDIs with midwives and 6 IDIs with hospital administrators. Table 1 presents the sociodemographic characteristics of service providers, and Table 2 presents the sociodemographic characteristics of women. Most women in this study were housewives or informal traders, Muslim and currently married. More than half of women had no formal education and had two or three children. All nurses and midwives were female (typical for Guinea), and most were less than thirty years old. All doctors were male, and most were less than 40 years old.

**Table 1:** Sociodemographic characteristics of participants: healthcare providers and administrators

	Nurse/midwives *n* = 13	Doctors *n* = 05	Administrators *n* = 06
Age (years)			
<30	6	1	0
30-39	3	3	0
40-49	2	0	1
≥50	2	1	5
Marital status			
Single	1	0	0
Married	12	5	6
Widowed	0	0	0
Gender			
Female	13	0	0
Male	0	5	6
Years of experience			
0-4	5	2	1
5-9	5	2	0
10-15	1	0	0
15+	2	1	5
Hospital location			
Urban	8	4	3
Peri-urban	5	1	3

**Table 2:** Sociodemographic characteristics of participants: women of reproductive age

	IDIs (*n* = 40)	FGDs (*n* = 8 FGDs^a^)
Age (years)		
<20	10	13
20-24	10	20
25-29	9	21
30-34	4	8
35-39	6	5
≥40	1	2
Marital status		
Single	0	1
Married	39	68
Divorced/Widowed	1	0
Location		
Urban	20	39
Peri-urban	20	30
Religion		
Christian	0	7
Muslim	40	62
Education		
None	18	36
Primary	6	11
Secondary	14	19
Tertiary	2	3
Diploma	0	0
Employment		
Civil servant	1	0
Hair dresser	0	3
Housewife	7	24
Tailor	5	15
Teacher	3	2
Trader	18	21
Students	6	4
Number of living children		
0-1	12	23
2-3	16	32
4-5	7	11
6+	5	3

^a^8 FGDs conducted with a total of 69 participants (6 FGDs conducted with 9 women, 1 FGD with 8 women and 1 FGD with 7 women)

This study explored how women are treated during childbirth in health facilities in Guinea, and this analysis focuses on perceptions and experiences of mistreatment of women during childbirth in health facilities, according to women and health workers. This topic was selected to explore the existence of mistreatment during childbirth in Guinea, given the lack of documentation. The results are presented according to the typology of mistreatment during childbirth in health facilities according to Bohren and colleagues [7]. The most commonly reported types of mistreatment in this study included verbal abuse, neglect and abandonment, and staffing constraints that affected how care was provided to women. This analysis of mistreatment experiences focuses on two key aspects of the phenomenon. First, to use the typology of mistreatment during childbirth to classify the different types of mistreatment that occur in Guinea and the participant’s reaction to such mistreatment. Second, this analysis describes women and providers’ views on the perceived factors influencing mistreatment, and suggestions for improvement in this context.

The majority of women in IDIs and FGDs reported positive childbirth experiences, but many women also reported situations that made them feel uncomfortable or unhappy during childbirth. Among women who reported uncomfortable experiences, there are two main narrations: (1) “lived experiences” where participants themselves experienced mistreatment during childbirth; and (2) “shared experiences” where participants detailed the experiences of mistreatment during childbirth from other women in their social circle. The importance of these “shared experiences” mistreatment during childbirth should not be underestimated, because these negative experiences are shared between friends and family members, and can influence other women’s attitudes and practices regarding their use of facility-based health services. Furthermore, understanding shared experiences is an important step to understanding the context of mistreatment during childbirth when such actions might be normalized within the health system and more easily accessible through third party testimony. Fourteen women in IDIs discussed experiences of mistreatment, where six women reported “lived experiences” and eight women reported “shared experiences”. In the FGDs, 27 uncomfortable experiences were shared, including 25 lived cases and two shared experiences.

Health workers felt generally satisfied about the services they provided; however, approximately one-third of health workers discussed mistreatment during childbirth: four “lived experiences” and two “shared experiences”. Healthcare providers spoke generally about care that they witnessed while working on the labor ward, rather than mistreating women themselves.

### Physical abuse

In Guinea, physical abuse during childbirth, such as slapping or pinching was considered unacceptable by women. Although physical abuse was not a common experience among participants in this study, two women reported “lived experiences” of being slapped or pushed by a service provider. For example, a woman reported witnessing a midwife physically abusing a woman during labor:

*Woman: …at my arrival the woman who was there was yelling at people that made unhappy. Even if I hear, I can’t say something. Once I went to the gynecologist for a visit, I show a woman who went for childbirth, suddenly I saw the midwife slapping the woman by yelling at her and her mother-in-law jumped on the midwife and slapped her till tearing her blouse. They struggled till they went out, you know, if you see something on your friends you don’t do anything. If not, everybody could get up and beat this midwife, we can’t repeat all that because it is not your child we have witnessed all that. If you see something happening to someone, you don’t know you can’t say anything*

*Interviewer: How did you feel?*

*Woman: I was upset, it seems it was my child who was beaten/hit.* [IDI woman, 39 years old, urban]

Observing this mistreatment was upsetting to the woman, and she felt powerless to intervene to protect the woman in labor. Women frequently described “shared experiences” where they had heard that other women who come to the facility for childbirth were hit by health workers. Some women believed that if they cried out during childbirth, they would be hit by the midwife: “…*they told me that in the hospital they often hit women…it seems when you cry during childbirth they hit you*”. These women believed that slapping a woman during childbirth was not helpful to encourage the woman to push, because they already understood the process of labor and delivery.

*Woman: I heard that there is a lady in the hospital there who slaps women who come to childbirth. If someone slaps me during my childbirth, I will pay back after because at that moment I don’t have force, because everybody knows how to give birth.* [IDI woman, 28 years old, urban]

In addition to slapping and hitting, several women described health workers pressing down with extreme force or sitting on their abdomen while they were in labor. While fundal pressure is used in some settings to accelerate the second stage of labor (although there is limited evidence for its use), the behavior described was characterized by women as particularly forceful and painful. In this study, women felt that this was a form of violence that they felt was “disturbing”, made them feel “weak”, and was often accompanied by insults from the provider.

*Interviewer: Could you please explain what makes them* [women in their community] *feel uncomfortable or unhappy?*

*Woman 4: They* [health workers] *press your belly and pull all sides till you become weak.*

*Woman 9: They were pressing my belly, they insulted and disturbed me.* [FGD women, urban]

*Woman: They* [health workers] *sit on your belly, a girl told me when she went to childbirth, they sit on her belly and her child didn’t survive.* [IDI woman, 24 years old, urban]

### Verbal abuse

Many women described childbirth experiences with verbal abuse from the health workers, including insults, yelling, discussing the woman’s intimate life, blaming, judgmental and accusatory comments, and threatening to throw a woman out of the health facility. Women also reported nonverbal insults or “whisperings”. In Guinea, a “whispering” is an expression of hard verbal unhappiness, where a sound is made by voluntary aspiration of air through closed teeth. Whispering is used to insult and shame someone, and such an act by a health worker is considered a grave offense against the woman. Women felt that they were “*gnermedé e hebdé konghudhi metudhi*” [“badly talked to by providers”] in several different circumstances. First, service providers grew frustrated and short-tempered with women when they did not follow instructions.

*Woman: That is why her practitioner doctor said she was difficult, but them too, they don’t do their work accurately. They insulted, her pulled her and yelled at her they did many things to her. Service providers were saying that she didn’t accept to keep calm in order to be treated.*

*Interviewer: Could you explain the situation?*

*Woman: Hum…Hum! What happened between them? This one I found her there, when I went to hospital…They said she went there since yesterday. At 4 p.m., but I found that she hadn’t childbirth. I found the woman disputing with service providers. If they tell her what is good for her, she refuses. It’s what nerves service providers. It’s how they insulted her till she gave birth at the same time with me, but it occurred that she had suffered a lot. She didn’t accept what service providers told her to do, but service providers mistreated her too. They yelled at her and insulted her.* [IDI woman, 19 years old, peri-urban]

Other women believed that when they cried out in labor, midwives reacted by yelling at them to keep quiet, rather than providing comfort. This woman describes a scenario where the midwife threatened to withhold care from her, because of her crying out from labor pains.

*Interviewer: Did you have any experience during your labor or during your stay at hospital after delivering that made you unhappy or uncomfortable?*

*Woman: When she found me on the bed, I was crying she told me to keep quiet, I cry too much. I replied “hay, no, I told her I can’t because since the day before yesterday I am having pains on my belly.” She told me that’s my business. She immediately took her bag and left the room. Since that time I didn’t talk to anyone. I was only crying and when she came back I didn’t look at her even though she finds me crying she went inside and I keep quiet.* [IDI woman, 22 years old, urban]

Women also described instances where they heard health workers “*soy soy*” [“murmur”] about their labor and childbirth experience, which disturbed and irritated the women. In the Guinea context, “murmurs” refer to health workers talking under their breath or reproaching women, and women viewed “murmurs” as health workers breaking their confidentiality by reporting to others about her private medical condition. Women described both “lived” and “shared experiences” of service providers insulting them, including mocking them for their sexual activity and even threatening to throw them out of the facility.

*Woman: Is the fact that they mistreat women…by telling her “we didn’t send you to get pregnant. While making love, you were not crying there, and here during childbirth you cry.” That is why I said I prefer to childbirth in men’s hands than women’s, because if you childbirth in men’s hands they take care of you, comfort you.* [IDI woman, 25 years old, urban]

*Interviewer: Could you explain why they feel unhappy or uncomfortable?*

*Woman 3: They didn’t handle me very well and were telling me violent words like if I was crying during the sexual relations, “Keep quiet, if not you leave here”.* [FGD woman, 23 years old, urban]

Such experiences sometimes prompted women to prefer a male provider over a female provider, because women believed that only female providers would make such comments.

Women felt embarrassed and humiliated by healthcare providers when things happened that were outside of their control, for example, when amniotic fluid or blood splashed on the provider. Providers responded to such situations by blaming the women for soiling their uniforms, which was distressing to women.

*Woman: As soon as the midwife ruptured the membranes, I didn’t know the water splashed at her, the midwife whispered, yelled and wanted to blame me. Honestly that act hurt and didn’t put me at ease and until now it is hurting in my heart. It is not my fault, I am not guilty. It’s the midwives who provoked that, breaking the water pocket.* [IDI woman, 33 years old, peri-urban]

Some service providers corroborated women’s experiences of mistreatment, including that other providers were verbally or physically abusive. These providers acknowledged that such mistreatment occurs, were upsetting for women to experience, and that women preferred to be treated with respect.

*Midwife: There are some women who report about mistreatment experiences that they met in health facilities. They say during their childbirth people yelled at them or they say among them this one is kind, but the other one is bad, when you go there they insult you and sometimes the even try to hit or beat you.*

*Interviewer: Why do you think this happened?*

*Midwife: If you see a wealth worker behaving like that, it’s because he doesn’t know his work. There a lack of competence, if you are competent and you know very well you job you can’t behave like that.* [IDI female midwife, 32 years old, peri-urban facility]

Health workers believed that women were verbally abused because of angry tempers, the health worker’s competence of managing women during childbirth, or because of a lack of cooperation from women.

### Painful vaginal exams

Women reported receiving frequent and painful vaginal examinations from different service providers, which was distressing to the woman as the examinations were conducted in front of other patients without any privacy, and providers did not explain why the examination was necessary. Administrators and health workers explained that in this setting, many service providers working on the maternity ward are underqualified and many trainees are present.

*Interviewer: Could you explain what makes her uncomfortable or unhappy?*

*Woman 5: When we go to childbirth, all the time they take their fingers and introduce them in us, whereas we are far from delivery yet, and before finishing, it occurs that your legs are hurting you and you are completely tired. All time they examine you and never tell you the truth, if you are far or not* [cervical dilation]*. They don’t tell you anything, only writing* [on medical records]*. If this one examines, she tells the other to come and see, I have examined and I found it like this you too come and see.*

*Woman 7: What we can keep in mind is that we don’t like the trainees. If you go there, each one comes, I don’t know if they want to examine you or if they are curious to see you, they don’t say what time you are going to deliver.* [FGD women, peri-urban]

Women disliked when examinations were conducted by trainees in particular, because they felt that the trainee would give the examination [“manipulation”] and leave without communicating the findings or progress to the woman.

### Neglect and abandonment

Women complained about neglect and abandonment throughout their encounter at the health facility, from reception through childbirth and discharge. Women felt neglected when health workers engaged in other tasks in the delivery room, when health workers took breaks to eat or sleep and when health workers played on their phones. As a result, women felt that they were “*musedè*” (suffering) alone until the baby came out.

*Woman: They* [women] *suffer during their labor and service providers do not look after them, because you are not up on the delivery table, that is why they do not look after you. It occurred in front of me, before I childbirth, they ask only if you are ready. If you are ready, you’ll go on the table, but if do not tell them you are ready, they will not look at you, and you will suffer because it is you which is hurting and you will suffer there till you deliver.* [IDI woman, 25 years old, urban]

Midwives also threatened to leave women alone during her delivery if they did not comply with their requests, such as to lie down on the delivery table and keep quiet. Women reported that midwives “*do not even look at you*” while you are in labor, rather than trying to help ease their pain.

*Woman 5: I will not go to that hospital, they abandoned me because I could not go up on the delivery table…when I came to childbirth my first child, I was suffering she told me to lie down, I couldn’t at that moment because the baby was between my legs, she told me if I don’t lie down she will go out and leave me, I told her there’s no problem…she told me if I childbirth on the floor she was going to abandon me and leave.* [FGD woman, peri-urban]

*Woman 1: Health workers I found there in the morning wanted me to childbirth, whereas it was not time so they abandoned me on the table and went to sit down.*

*Woman 5: …I stayed alone in the childbirth room till it was nearly time to childbirth there was no one beside in the childbirth room.* [FGD women, urban]

In some cases, women gave birth in the health facility without the presence of a skilled provider. Women described situations where childbirth took place without service providers present, and where some were abandoned and threw away their gloves by saying that women did not respect their instructions.

*Woman: I have had an experience during my childbirth which made me unhappy…they didn’t take care of me and I didn’t have anybody beside, I suffered a lot. They went to lie down until I delivered alone.* [IDI woman, 25 years old urban]

Women reported “shared experiences” that their friends may not go to the hospital for childbirth in the future after feeling neglected and abandoned a previous childbirth. This neglect seems to occur when providers engage in other activities or if they take a break to relax. In both scenarios, the provider is not present when the woman needs them. However, health workers disagreed that women were neglected or abandoned in their facilities. They believed that women might feel neglected if they were being uncooperative during labor, rather than from bad behavior from the health worker. They recognized that the consequences can be drastic and can even lead to the baby’s death.

A nurse explained that some Fulani women deliver without intervention from health providers, and often labor quietly and without agitation. In contrast, other women need more support to progress through labor well, particularly primigravidas. Health workers believed that the woman should be prepared for what to expect during labor and childbirth during antenatal care, so that upon arrival in the health facility, they are prepared both mentally and physically.

*Interviewer: Sometimes women are mistreated or poorly treated during childbirth. Have you ever seen or heard of this type of mistreatment happening in your work? Could you explain the situation?*

*Midwife: Yes, it occurs. I said the Fulani woman is very correct…she can give birth without being assisted, she is never agitated, she doesn’t cry or yell. But there are some women who come to childbirth, primigravadas, sometimes if they come, you have to tell them how it [labour] is going to occur…because if she is on the table, she says she doesn’t want to give birth there…because she was not informed of the different stages* [IDI female midwife, 57 years old, peri-urban facility]

However, there appeared to be a disconnect between what was supposed to be communicated during antenatal care visits and what knowledge women retained when they arrived at the health facility. During labor, if health workers do not educate and communicate to women about the different stages of labor and what to expect, then the women may be less likely to listen to the health workers’ requests. This can frustrate the health worker, and in some cases may lead to the health worker threatening to leave the woman to deliver alone.

### Poor physical conditions of the facility

Both providers and women describe the study health facilities as characterized by unfavorable physical conditions, including poor hygiene, presence of mosquitoes due to lack of window screens and doors, and insufficient number of beds. Providers explained that during periods of overcrowding, women may have no other option than to deliver on the floor. Furthermore, the delivery room lacks running water, thus requiring the woman’s accompaniers to go and fetch water for needs related to childbirth. These insalubrious conditions are a source of unhappiness and discontent for women arriving to the hospital for delivery.

*Interviewer: Have you had any experience during your labor or stay in hospital after delivery which made you unhappy or uncomfortable?*

*Woman: Only two things made me unhappy and those ones are filthy and mosquitoes…I had just delivered… I was lying there and mosquitoes were biting me, it was filthy, and the mosquitoes were biting me.* [IDI woman, 19 years old urban]

Midwives corroborated women’s stories about delivering on the floor of the labor ward. They suggest that this happens infrequently, and predominantly results from health system deficiencies, such as the lack of delivery beds, rather than maliciously on behalf of the providers.

*Midwife: In the delivery room sometimes women come in 2, 3 or more than 5 whereas there only two tables in the delivery room, so some deliver on the floor and it leads to women’s nervousness. It is not good for her, and the woman will say she has been mistreated.* [IDI female midwife/nurse, 23 years old, urban facility]

### Staffing constraints

Due to staffing constraints, health facilities frequently call on trainees to provide care, and often the trainees make up the majority of health workers on duty, particularly in peri-urban and rural areas. Their often unsupervised presence is a source of anxiety for many women, due to their young age, lack of personal childbirth experience, and lack of professional experience. Some women lamented that trainees have less compassion and empathy and are more hot-tempered towards women in labor, compared to qualified providers. Women resented that trainees talked to them rudely and did not explain processes or procedures. One woman went so far as to say that the presence of trainees was a reason why some women chose to deliver at home. Given the staffing constraints, the trainees may not have supportive supervision to continue their education, and may be quick to send women for caesarean section, rather than cope with the process of labor.

*Woman 7: What we can keep in mind in that, is we don’t like the trainees, if you go there each one comes I don’t know if they want to examine you or if they are curious to see you, they don’t say what time you are going to deliver. If it is midwives most of the time they tell you the exact time you are going to childbirth…that is why I said a trainee is not going to manipulate me, I completely refused if the responsible does not come to examine me, trainees are not going to examine me…Trainees talk badly, they don’t tell you anything, it is only to manipulate and leave you there. And they are girls who have never given birth, young girls who have never delivered who come to examine you, manipulate you…*

*Woman 6: Trainees badly talk to people, maybe it’s in their habit. Many women say that trainees talk badly, those who work badly talks to people…*

*Woman 3: It is the same thing if you go to the hospital you want to childbirth, if you do not deliver in 30 minutes they send you to the surgical room. Yes, yes, if you stay a bit longer they send you to the surgical room because those who are there are trainees. They say you can’t deliver naturally.* [FGD women, peri-urban]

On the other hand, other women think that older midwives are slow and must be replaced by young health workers.

*Woman 3: I accompanied a friend of mine to childbirth, we found an old lady as service provider, who in case of emergency can’t run quickly to assist women, so they ought to be replaced by young health workers, because if it is a young person she or he can run quickly, old ladies do not refuse but they are tired.* [FGD woman, 24 years old, peri-urban]

### Unreasonable requests of women by health workers, bribery and extortion

Administrators explained that in Guinea, maternal and neonatal care is subsidized by the government, and the woman is not expected to pay for services. Officially, the government provides hospitals with maternity wards with delivery kits free-of-charge, which includes cotton, gloves, plastic sheets, and other supplies needed for a delivery, and the hospital administration gives the government delivery kits directly to women. However, in practice there are sometimes stock-outs, and health workers may use that opportunity to request necessary materials from women and their families. Furthermore, women also reported being detained in the health facility, unable to leave until they pay their bills or bribes.

*Woman: …they made wait for a long time when I finished delivering due to money and harsh words from trainees.*

*Interviewer: …you ought to tell all from the beginning to the end, who made you feel unhappy?*

*Woman:…it’s mosquitoes biting and the fact that they made wait for a long time in the morning due to money payment.…When I finished* [delivering the baby] *I didn’t have money and I told them to let me go home and I will give their money to a child who will bring it to them; but they didn’t accept. My grandmother was obliged to go home and fetch their money after that they released us…they said were not going home if we haven’t paid the “thiogou saboundè”, I mean money to be paid cash due to the childbirth assistance. It’s a bribe money! I told them to let me go home I will send the money. They refused. It’s only when I sent the money they released me.* [IDI woman, 26 years old, urban]

When bribes or informal payments to providers were made, the type of care provided to the woman depends on the size of the payment made by the family to the provider. Furthermore, women perceived that because childbirth services were free at the hospital, the providers would not be responsive to their needs.

*Woman: Yes I heard that when you don’t give money they will not help you…Yes she went and they told her to do all herself so she was abandoned on the table she suffered and finally she came down on the floor to childbirth, crying and they were badly talking to her by insulting her and yelling at her till she childbirth naturally…And the health worker she found there didn’t take care of her because delivering is free of charge…when you go to hospital you have to pay money or they do not really take care of that woman.* [IDI woman, 33 years old, urban]

An administrator explained a situation in which a woman was forced to pay a bribe to the healthcare providers in his health facility:

*Administrator: Yes, there are some cases [of bribery]. In what I know, it happened in December 2014. We’ve even received complaints. But we have here a disciplinary board, when there is such problem, we gather everybody to draw lessons. I think that it is about money…a woman that came from Sangareah, a village, very far from here in Pita District. Firs,t the reception was not good, providers that were there told the woman that she delayed too much. So they asked her for many money for her first care. The supervisor (Head of Maternity) of the guilty health workers has been congratulated because he punished those [health workers] who did it. In fact, it is matter of mistreatment…Fortunately, there was no death* [IDI male general supervisor, 46 years old, suburban facility]

### Reactions to mistreatment

Women’s reactions to experiencing mistreatment during childbirth can be categorized into three types of responses: (1) acceptance and forgiveness; (2) retaliation against the provider; and (3) changes to future care-seeking behaviors. First, it is important to note that women who experienced mistreatment may also express satisfaction with their childbirth experience, particularly if they had a live baby, because they viewed a live baby as the most important outcome. These women forgive the providers for mistreating them, either because they believe that the mistreatment helped them to deliver a healthy baby or because acceptance and forgiveness is viewed as their only option. Furthermore, some women believe that according to customs and tradition in Guinea, the age and education differences between health providers and women prevents women from speaking out against their poor treatment. Others believe that although they were poorly treated, the situation was not serious and forgiveness is necessary to move forward.

*Woman 7: I wasn’t pleased, some providers are older than us and others, we are the same age. We can’t insult or beat them what we can is to tell them the truth…*

*Woman 3: It’s harmful, but we have to forgive.*

*Woman 9: I wasn’t pleased, but when I saw your baby in good condition, you will forget all…*

*Woman 6: When they harm you, you ought to forgive because you can neither beat them nor insult.* [FGD women, urban]

However, other women felt angry with the providers after being mistreated, and considered retaliation or revenge against the providers: “*I will wait until after delivery, I’ll get revenge* [FGD woman, 24 years old, urban]

Finally, some women were so upset with how they were mistreated, that they would not go to the same hospital again for future deliveries. Given that there are few options for other nearby hospitals with maternity wards, some women may therefore choose to deliver at home: “*I was angry* [at the mistreatment] *because if there isn’t any hospital except this one, I prefer delivering at home.* [FGD woman, 24 years old, sub urban]. Expectations of mistreatment at the facility was also cited by women as a reason why women choose to give birth at home:

*Interviewer : Why do women in this community deliver at home?*

*Women 3 : …There are many trainees, who [women] say abuse them, they don’t show respect to them. It is the reason they don’t go to the hospital. Therefore, they prefer delivering at home to avoid those trainees.* [FGD woman, 29 years old, urban]

### Perceived factors that influence mistreatment during childbirth

All participants (women, providers, administrators) were asked what they perceived to influence mistreatment during childbirth. These factors are related to four main categories: (a) essential physical resources; (b) health system and workforce; (c) service providers’ attitudes and practices; and (d) women’s characteristics and behavior.

Administrators, service providers and women all recognized that medicines and equipment (such as tables, beds, ultrasound machines) are insufficient or do not exist at all in the maternity ward. Inadequate and unreliable sources of water and electricity also contribute to a stressful working environment, consequently causing health workers to express their frustrations at women. Administrators and providers explained that as with many low- and middle-income countries, Guinea faces a health worker shortage, and the insufficient number of skilled health workers means that health workers are often overworked. As a result, women may be inadequately managed during labor, because there are not enough health workers to provide quality care.

Women frequently blamed experiences of mistreatment on the attitudes and behaviors of the health workers, believing that they were poorly trained at their jobs and overworked. In particular, women blamed trainees and younger midwives for bad attitudes and behaviors. Likewise, some service providers attribute this bad behavior due to insufficient training and a heavy workload. However, administrators believe that these service providers do not respect professional norms and procedures of maternity services, which leads to mistreatment.

Sometimes, service provider’s behavior influences how women react. As a result, women may become upset, disturbed, or disobedient, which is further exacerbated by their lack of psychological support and lack of pain relief. Providers consequently feel that women are disobeying their advice and acting obstinate, which further contributes to the providers’ stress and frustration. In some cases, women lashed out at health workers when they were in pain or feeling that they were treated poorly, and health workers felt demoralized and demotivated to provide care.

### Suggestions to improve how women are treated during childbirth

At the end of the IDIs and FGDs, participants suggested several solutions to prevent mistreatment from occurring. These solutions are presented in Table 3 and are grouped according to: (1) solutions at the health facility and health system levels; (2) solutions at the service provider level; and (3) solutions related to the woman. At the health facility and system levels, participants focused the solutions on improving supply chains, physical structures of the health facilities, and improving water, sanitation and electricity. Restructuring and repairing these physical resources would help to improve the physical environment of the health facility. Participants also highlighted the need for improvements to the number of qualified and component skilled providers, who were motivated to provide high quality care. This included enhanced training programs for physicians, midwives and nurses, as well as prompt and regular payment of improved salaries and consideration of interventions to improve provider motivation (e.g.: pay-for-performance). At the level of the woman, participants suggested improving how information about the process of labor and childbirth is communicated to women, for example during antenatal care visits, the use of sketches and images, as well as through television and radio. Finally, participants suggested the creation of a platform to exchange ideas between women, communities, providers and administrators on improving quality of care and accountability.

**Table 3:** Participants’ suggestions to improve how women are treated during childbirth

Solutions related to the women	Solutions at the service provider level	Solutions at the health facility and system level
Improve sensitization of women during antenatal care through adequate counselling, to better prepare women for what to expect during childbirth, as well as sensitization on the radio and television, and through sketches and images.	Create equity for women accessing childbirth care: effective application of free-of-charge maternity care, to avoid discrimination according to women’s income.	Increase the number of qualified and competent skilled service providers: to better plan the activities and offer a quality of care, and to reduce workload.
Create a platform of idea exchange between women, families, service providers, and administrators in order to better address women’s concerns, particularly regarding mistreatment during childbirth	Improve the quality of and management of women: conduct training and sensitization programs for providers to better take care women, focused on improving interpersonal skills, coping with stress and effective communication.	Improve supply chains to ensure consistent medical and drug supplies: to mitigate delays in receiving appropriate care
	Increase motivation of health workers such as pay-for-performance and improved salaries to encourage them to provide good quality of care.	Improve physical resources, including refitting labor rooms and increase the number of hospital beds: to avoid childbirth on the floor and promote privacy and labor companionship.
		Repair the water and electricity supply system in health facilities as well as reinforce hygiene in the unit. Such practices will help to maintain the area clean and avoid requesting women who go there to childbirth to bring water for their needs.

## Discussion

This is the first known study on mistreatment during childbirth in Guinea, and the results suggest that mistreatment during childbirth in Guinea is a reality. Women in this study shared their own experiences or the experiences of women they knew about being mistreated during childbirth. This mistreatment included physical abuse such as slapping, pinching and excessive fundal pressure. Women also experience verbal abuse, neglect and abandonment during childbirth. Some women justified mistreatment if they were uncooperative; for example, it was acceptable to some women that a provider slaps her, if it is to save her life or the life of her baby. This suggests that some women may have the perception that there is a medical justification for slapping a woman, as they believe that these acts are intended to “help” the woman to push the baby out. Further inquiry is needed to explore how such acts of mistreatment relate to medical harm, harmful practices, and the provision of “interventions” without evidence. Maternity wards in Guinea have severe deficits of skilled and competent providers. For example, there are roughly 18 physicians and 18 nurses/midwives per 100,000 people in Guinea [17]. The health workforce crisis is further exacerbated by inequitable dispatching of health workers between urban and peri-urban/rural areas and the refusal of health workers to leave Conakry.

The WHO framework for quality of care for pregnant women highlights the importance of improving women’s experiences of care, and recognizing that the participation of women in healthcare programs is critical to improving services [6]. This means treating women with respect and dignity, communicating effectively between the provider and woman, and providing emotional support to the woman [6]. Results from our study support the importance of using a holistic view to improve quality of care and reduce mistreatment during childbirth, by highlighting women’s experiences of childbirth. Similarly, a systematic review by Bohren and colleagues explored facilitators and barriers to women giving birth in health facilities and concluded that women’s perceptions of the quality of care may influence her healthcare choices, including the decision to deliver at home [18]. Results from our study also suggest that both women’s own experiences of poor quality care, and experiences of their friends or family may influence a woman’s future healthcare decisions.

Limited research has been conducted in West Africa on improving women’s experiences of care. However, a study conducted by Fujita and colleagues in Benin showed that promoting a supportive environment for women during childbirth can improve women’s experiences of care [19]. The supportive care model included improved communication between women, their families and providers, encouraging women to make informed decisions about their care, allowing women to give birth in a position of their choice, and allowing women to have labor companions [19]. This model of supportive care may be helpful to consider implementing in Guinea, as it is a low-cost intervention that may greatly improve women’s experiences of care and encourage women to give birth in health facilities.

### Limitations and strengths

This study has some weaknesses and some strengths. Although the study sites were minimally impacted by Ebola, during the epidemic many communities associated health professionals or discussions about health with Ebola. Therefore, recruiting participants was sometimes challenging, as some husbands refused to allow their wives to participate in the study. However, the study team worked to dispel these fears by reassuring potential participants that this study was related to childbirth, and would not put them at risk of Ebola. Interviews were often conducted in local languages (Pular and Malinke), and transcription from local language to French for analysis was sometimes challenging. Although this study was conducted in two prefectures in one region in Guinea, we believe that the results may be transferable to other settings in Guinea. This is because Mamou is an urban center, and attracts people migrating from different parts of Guinea, and also that anecdotal reports from other prefectures of Guinea suggest that the same mistreatment is happening there. Future research could explore if this hypothesis is correct. Freedman and colleagues suggest that understanding the experiential level of mistreatment is an important starting point, but that in settings where mistreatment is common, both women and providers may not view mistreatment as a violation [20]. Aligned with this supposition from Freedman and colleagues, we asked participants generally about any experiences that made them feel unhappy or uncomfortable, then asked about specific actions that Bohren and colleagues categorized as mistreatment during childbirth [7]. This dual approach allowed us to explore both individual and shared experiences, as well as social norms influencing mistreatment. Despite the weaknesses, this is the first study on the topic of mistreatment during childbirth in Guinea and the results are important to improving maternal health. Furthermore, there is limited published research on maternal health from Guinea; thus this study makes a contribution to the international academic literature. Finally, the research team was composed of both sociologists and medical professionals, which is necessary to explore and understand this complex topic of mistreatment.

### Research implications and conclusions

Moving forward, the authors will facilitate a dissemination workshop to share the results of this study with the Ministry of Health, WHO-Guinea, health facilities, faculty of medicine and other partners. The goal of this workshop is to recommend strategies to include the prevention of mistreatment during childbirth into national and prefecture-level strategic plans and increase awareness. Results will also be shared with the communities to validate the results and explore how to design interventions to prevent mistreatment during childbirth. Future research in Guinea should focus on measuring how often mistreatment during childbirth occurs and what interventions or programs can be developed to change the behavior of providers to prevent mistreatment from occurring. We hope that this study will influence other researchers around the world to conduct similar studies in their context.

## Conclusions

In conclusion, this study shows that mistreatment exists in Guinea and occurs in many forms, including physical and verbal abuse, neglect and abandonment. We recommend that stakeholders in the Ministry of Health, WHO, and hospitals in Guinea start to discuss this important topic and work together to prevent mistreatment from occurring.

## Abbreviations

CERREGUI: Cellule du recherche en la sante de la reproduction
FGDs: Focus group discussions
HRP: World Health Organization Human Reproduction Programme
IDIs: In-depth interviews
MDGs: Millennium Development Goals
MMR: Maternal mortality ratio
RP2: Research Project Review Panel
